# Molecular detection of human Plasmodium species in Sabah using PlasmoNex™ multiplex PCR and hydrolysis probes real-time PCR

**DOI:** 10.1186/s12936-015-0542-5

**Published:** 2015-01-28

**Authors:** Ping Chin Lee, Eric Tzyy Jiann Chong, Fread Anderios, Yvonne AL Lim, Ching Hoong Chew, Kek Heng Chua

**Affiliations:** Faculty of Science and Natural Resources, Universiti Malaysia Sabah, Jalan UMS, 88400 Kota Kinabalu, Sabah Malaysia; Diseases Section, Sabah State Public Health Laboratory, Kota Kinabalu, Sabah Malaysia; Department of Parasitology, Faculty of Medicine, University of Malaya, 50603 Kuala Lumpur, Malaysia; Faculty of Health Sciences, Universiti Sultan Zainal Abidin, 21300 Kuala Terengganu, Terengganu Malaysia; Department of Biomedical Science, Faculty of Medicine, University of Malaya, 50603 Kuala Lumpur, Malaysia

**Keywords:** *Plasmodium* species, Multiplex PCR, PlasmoNex™

## Abstract

**Background:**

Malaria is a vector borne-parasitic disease transmitted through the bite of the infective female *Anopheles* mosquitoes. Five *Plasmodium* species have been recognized by World Health Organization (WHO) as the causative agents of human malaria. Generally, microscopic examination is the gold standard for routine malaria diagnosis. However, molecular PCR assays in many cases have shown improvement on the sensitivity and specificity over microscopic or other immunochromatographic assays.

**Methods:**

The present study attempts to screen 207 suspected malaria samples from patients seeking treatment in clinics around Sabah state, Malaysia, using two panels of multiplex PCRs, conventional PCR system (PlasmoNex™) and real-time PCR based on hydrolysis probe technology. Discordance results between two PCR assays were further confirmed by sequencing using 18S ssu rRNA species-specific primers.

**Results:**

Of the 207 malaria samples, *Plasmodium knowlesi* (73.4% *vs* 72.0%) was the most prevalent species based on two PCR assays, followed by *Plasmodium falciparum* (15.9% *vs* 17.9%), and *Plasmodium vivax* (9.7% *vs* 7.7%), respectively. Neither *Plasmodium malariae* nor *Plasmodium ovale* was detected in this study. Nine discrepant species identification based on both the PCR assays were further confirmed through DNA sequencing. Species-specific real-time PCR only accurately diagnosed 198 of 207 (95.7%) malaria samples up to species level in contrast to PlasmoNex™ assay which had 100% sensitivity and specificity based on sequencing results.

**Conclusions:**

Multiplex PCR accelerate the speed in the diagnosis of malaria. The PlasmoNex™ PCR assay seems to be more accurate than real-time PCR in the speciation of all five human malaria parasites. The present study also showed a significant increase of the potential fatal *P. knowlesi* infection in Sabah state as revealed by molecular PCR assays.

## Background

Malaria is a mosquito-borne parasitic disease cause by the unicellular, eukaryotic protozoan parasites of the genus *Plasmodium* and the infective female *Anopheles* mosquitoes are the sole vector of human-to-human transmission. Malaria continues to be one of the most severe global public health problems that affect many of the tropical and subtropical poorest nations. Five causative *Plasmodium* parasites have been recognized by World Health Organization (WHO) as able to infect humans [1].

Malaysia is situated in the hot, humid equatorial region and, therefore, is receptive and vulnerable to the transmission of malaria. The malaria main focal regions in Malaysia include Sabah and Sarawak states situated on the Borneo Island and central interior regions of Peninsular Malaysia. These areas are also the home for a majority of the isolated indigenous populations. Despite significant reduction in malaria cases over the centuries, the surge of *P. knowlesi* infections across Malaysia, especially Malaysian Borneo, poses a challenge to malaria control programmes, which aim to eliminate malaria in Peninsular Malaysia by 2015 and in Malaysian Borneo by 2020 [1,2].

The empirical clinical diagnosis remains the most common method to diagnose malaria that is based on the observation of the clinical features of the disease. However, the accuracy of this clinical presumptive diagnosis is poor due to extremely wide spectrum of clinical signs and symptoms ranging from mild to severe malaria. Basically, microscopy (parasite morphology identification), immunochromatographic-based rapid diagnostic test (antigen detection), and molecular PCR assays (parasite nucleic acid detection) are the three main malaria diagnostic methods and they target the parasites in the peripheral blood with wide ranges of sensitivity and specificity as reviewed by Moody [3].

Overall, the advent of molecular PCR-based diagnostics has produced higher specificity and sensitivity in the identification and differentiation of all five human malaria parasites up to species levels. As a whole, the currently described molecular nucleic acid amplification PCR assays can be subdivided into three categories, there are: i) conventional-based PCR assays, such as nested PCR [4-6], semi-nested PCR [7], and single step multiplex PCR [8,9], ii) real-time or quantitative PCR (qPCR) assays based on fluorescence dyes (SYBR Green, high resolution melting) or hydrolysis probes technologies [10-12], and iii) the simplest and least technically demanding loop-mediated isothermal amplification (LAMP) assay [13]. Overall, PCR is able to detect parasites at low titer, generally below 5 parasites/μl of blood for all five human *Plasmodium* parasites [3,7-9,11]. Amongst these molecular assays, nested PCR [4-6] targeting 18S ssu rRNA genes of all five human malaria parasites has been considered the molecular gold standard for malaria detection. However, due to the cumbersome and multiple amplification reactions that are needed in nested PCR assay (at least six PCRs conducted to differentiate all five human *Plasmodium* species), many researchers have attempted to develop a simpler, single step multiplex PCR system, which allows simultaneous identification of malaria parasites in a single tube reaction [8-12]. Multiplex PCR undoubtedly shorten the time and may be a useful diagnostic adjunct for diseases, such as malaria, that require prompt and effective treatment.

In the present study, 207 patient samples suspected for malaria were screened using two multiplex PCR assays both targeting 18S ssu rRNA gene of human *Plasmodium* species, single-step multiplex PCR assay (PlasmoNex™) [9] and combinations of two real-time PCR assays based on hydrolysis probes technique [10,12,14], respectively. Due to the lack of *P. ovale* case in Malaysian scenario, real-time primers and probe specific for this species was precluded in the present study. The results obtained from two PCR assays (PlasmoNex™ PCR and real-time PCR) were then compared. The discordances at the species level of two PCR assays were then confirmed by DNA sequencing. Overall, the aim of the present study was to test the application of these two published multiplex PCR platforms in the clinical diagnosis of malaria disease.

## Methods

### Study site and sample collection

The 207 clinical suspected malaria blood samples for the present study were collected between June 2012 and January 2013 from patients seeking medical care at government clinics around Sabah state, Malaysia. Approximately 3 ml of whole blood were collected in EDTA tube. Standard Giemsa-stained thick and thin blood films were prepared in the field and *Plasmodium* infection was determined by a field microscopist and then sent together with blood tubes to Sabah State Health Department. Genomic DNA was extracted from 200 μl of blood sample using QIAamp DNA Mini Kit (Qiagen, Germany), accordingly to manufacturer’s instructions.

### Hexaplex PCR (PlasmoNex™)

Multiplex PCR was carried out as described by Chew *et al.* [9]. Generally, 15 μl of PCR reagent mixture containing 20 mM of Tris–HCl, 20 mM of KCl, 5 mM of (NH_4_)_2_SO_4_, 3.0 mM of MgCl_2_, 0.2 μM of each dNTP, pooled primers mixture, 1 U of Maxima® Hot Start Taq DNA polymerase (Thermo Scientific, USA), and 1.5 μl (~10 ng) of template DNA were used in the detection study. PCR amplification was carried out with an initial denaturation step at 95°C for 5 min; 35 repeated cycles at 95°C for 30 sec, 56°C for 30 sec, 65°C for 40 sec, followed by a final extension at 65°C for 10 min using Mastercycler® Gradient 5331 (Eppendorf, Hamburg, Germany). The amplified products were visualized on ethidium bromide stained 3% (w/v) agarose gel (Promega, Madison, WI) and gel image was captured using Gel Doc™ 2000 Gel Documentation System (Bio-Rad, USA).

### Real-time PCR

Real-time PCR was performed by using primers, probes, and reaction conditions described previously by Shokoples *et al.* [12] and Divis *et al.* [14] with the following modifications: fluorophores for probes of *P. falciparum* were changed to Cy5-BHQ-1 and *P. vivax* to Texas Red-BHQ-2. Primers and probes were synthesized by Bioneer Corporation (South Korea) and are listed in Table 1 with the respective concentrations for each reaction. Three separate reactions were performed: (1) a screening reaction for the presence of *Plasmodium* species with *Plasmodium* genus-conserved primers pair (Plasmo1 and Plasmo2) and the Plasprobe to detect a conserved region of the *Plasmodium* 18S ssu rRNA gene of all five human malaria parasites [10], (2) a multiplex PCR for the detection of three *Plasmodium* species, i.e., *P. falciparum*, *P. vivax*, and *P. Malariae,* using species-specific forward primers paired with Plasmo2, and species-specific probes [12], and (3) a monoplex PCR for the detection of *P. knowlesi* with Plasmo1, Plasmo2 primers and a Pk probe [14]. Briefly, the monoplex and multiplex assays for *Plasmodium* speciation were performed with a final volume of 25 μL containing 5 μL of template DNA, 12.5 μL QuantiFast Multiplex PCR master mix (Qiagen, Germany), and 7.5 μL of pooled primers and probes mix. All assays were performed under standard conditions (1 cycle of 95°C for 5 mins; 45 repeated cycles of 95°C for 30 sec and 60°C for 30 sec) with the CFX96 Real-time PCR machine (Bio-Rad, USA). A cut-off of 40 cycles was used to define positive samples. The test panel included a number of controls: negative sample extraction as a negative control, β2-macroglobulin (β2M) target amplification as a positive extraction control for the sample and a positive reference control to detect any variation between runs and non-template control for each of the master mixes.

**Table 1:** **Primers and probes used for screening and identification of** ***Plasmodium*** **species**

**Species**	**Primer or probe**	**Conc. [nM]**	**Sequence (5’ → 3’)** ^**b**^	**References**
*Plasmodium* spp.	Plasmo1	200	GTTAAGGGAGTGAAGACGATCAGA	[10]
*Plasmodium* spp.	Plasmo2	200	AACCCAAAGACTTTGATTTCTCATAA	
*Plasmodium* spp.	Plasprobe	50	*FAM*-ACCGTCGTAATCTTAACCATAAA	
			CTATGCCGACTAG-*BHQ-1*	
*P. falciparum*	Fal-F primer	200	CCGACTAGGTGTTGGATGAAAGTGTTAA	[10,12]
*P. falciparum*	Falcprobe^a^	80	*Cy5*-AGCAATCTAAAAGTCACCTCGAA	
			AGATGACT-*BHQ-1*	
*P. vivax*	Viv-F primer	50	CCGACTAGGCTTTGGATGAAAGATTTTA	
*P. vivax*	Vivprobe^a^	80	*TR*-AGCAATCTAAGAATAAACTCCGA	
			AGAGAAAATTCT-*BHQ-2*	
*P. malariae*	Mal-F primer	50	CCGACTAGGTGTTGGATGATAGAGTAAA	
*P. malariae*	Malaprobe	50	*FAM*-CTATCTAAAAGAAACACTCAT-*MGBNFQ*	
*P. knowlesi*	Pkprobe	80	*FAM*-CTCTCCGGAGATTAGAACTCTTA	[14]
			GATTGCT-*BHQ-1*	
Human	β2M-F	900	TGAGTATGCCTGCCGTGTGA	[15]
Human	β2M-R	900	ACTCATACACAACTTTCAGCAGCTTAC	
Human	β2M-probe	100	*FAM*-CCATGTGACTTTGTCACAGCCCA	
			AGATAGTT-*TAMRA*	

^a^Probe sequence is as previously published [11], with modified fluorophores.

^b^TAMRA, 6-carboxytetramethylrhodamine; MGBNFQ, minor groove binding nonfluorescent quencher; BHQ, black hole quencher; Cy5, cyanine; FAM, carboxyfluorescein; TR, Texas Red.

### Sequencing

Sequencing was only performed on the samples for which PlasmoNex™ and real-time PCR gave different speciation results. Sequencing was carried out with ABI Prism BigDye terminator cycle sequencing kits and ABI Prism 310 automated sequencer (Applied Biosystems, USA). Sequencing results were then BLAST searched on GenBank database for species determination.

### Diagnostic sensitivity and specificity for three species

The diagnostic sensitivity (true positive rate), specificity (true negative rate), positive predictive value (PPV) (probability that the diseases is present when the test is positive), negative predictive value (NPV) (probability that the diseases is not present when the test is negative), and disease prevalence (DP) of three species, i.e., *P. vivax*, *P. falciparum*, and *P. knowlesi* were calculated, based on 207 malaria positive samples, using PlasmoNex™ as the standard. The 95% confidence interval (95% CI) was also calculated using MedCalc-Diagnostic test evaluation [16]. The calculations were expressed as percentage for ease of interpretation.

## Results

### PlasmoNex™

Of the 207 samples analyzed, 20 (9.7%), 33 (15.9%), and 152 (73.4%) samples were identified as single infection of *P. vivax*, *P. falciparum*, and *P. knowlesi* respectively, whereas two (1.0%) samples were examined as triple-species mixed infections of *P. vivax*, *P. falciparum*, and *P. knowlesi*, which were further confirmed via sequencing. Neither *P. malariae* nor *P. ovale* were detected amongst the samples (Table 2).

**Table 2:** **Comparison of diagnosis of** ***Plasmodium*** **species by PlasmoNex™** **PCR and hydrolysis probes real-time PCR for the sample collected from Sabah (n = 207)**

***Plasmodium*** **spp**		**No. of cases identified by PlasmoNex™** **(%)**				
		***P. vivax***	***P. falciparum***	***P. knowlesi***	***P. vivax*** **+** ***P. knowlesi*** **+** ***P. falciparum***	**Total**
No. of cases identified by species-specific real-time PCR* (%)	***P. vivax***	16 (7.7)	0	0	0	16 (7.7)
	***P. falciparum***	0	33 (15.9)	2 (1.0)	2 (1.0)	37 (17.9)
	***P. knowlesi***	0	0	149 (72.0)	0	149 (72.0)
	**No speciation**	4 (1.9)	0	1 (0.5)	0	5 (2.4)
	**Total**	20 (9.7)	33 (15.9)	152 (73.4)	2 (1.0)	207 (100.0)

*Real-time PCR speciation results were based on species-specific hydrolysis probes.

### Real-time PCR

Real-time PCR results indicated that all 207 malaria samples were positive with *Plasmodium* infections based on genus-conserved primers and probe, i.e., Plasmo1, Plasmo2, and Plasprobe. Species-specific real-time PCR indicated that 202 malaria samples were caused by single-species infection, i.e., 16 (7.7%), 37 (17.9%), 149 (72.0%) by *P. vivax*, *P. falciparum*, and *P. knowlesi*, respectively, while determination up to species level based on species-specific primers and probes failed for the balance five samples. No *P. malariae* infection was detected based on real-time PCR assay (Table 2).

### Sequencing result

Nine discordant results between two PCR assays were further confirmed via sequencing using 18S ssu rRNA species-specific primers. Four and one samples that failed in speciation based on species-specific real-time PCR primers and hydrolysis probes were actually single infected sample of *P. vivax* and *P. knowlesi*, respectively. Two samples diagnosed as *P. falciparum* infection based on multiplex real-time PCR assay were actually infected with *P. knowlesi* based on sequencing results and BLAST data, which were in agreement with the results obtained from PlasmoNex™ assay. Another two Falcprobe positive samples, which were suspected with mixed infections based on PlasmoNex™ results were then sent for sequencing and further confirmed that both samples were actually triple-species mixed infections with *P. falciparum, P. vivax*, and *P. knowlesi*. All mentioned sequencing results (n = 9) were in agreement with the results obtained from PlasmoNex™.

### Diagnostic sensitivity and specificity for three species

The sensitivity and specificity of the real-time PCR in detecting *P. vivax*, *P. knowlesi* and *P. falciparum* were 72.7% and 100%, 96.8% and 100%, and 100% and 98.8%, respectively in species diagnosis. For *P. falciparum* positive samples, the probability of detection using the real-time PCR was 94.6% but the probability to not detect the *P. vivax* and *P. knowlesi* was 96.9% and 91.4%, respectively, in those negative samples when compared to PlasmoNex™. This indicated that *P. knowlesi* (74.4%) was the most prevalent among all *Plasmodium* species, followed by *P. falciparum* (16.9%) and *P. vivax* (10.6%) in Sabah (Table 3).

**Table 3:** **The sensitivity, specificity, positive predictive value (PPV), negative predictive value (NPV), and disease prevalence (DP) of the real-time PCR compared to PlasmoNex** ^**TM**^ **PCR**

**PlasmoNex™** **as standard (Confirmed by sequencing)**							
**Species**		***P. vivax***		***P. falciparum***		***P. knowlesi***	
Test*		**Positive**	**Negative**	**Positive**	**Positive**	**Negative**	**Positive**
**Species-specific real-time PCR**	**Positive**	16	0	35	2	149	0
	**Negative**	6	185	0	170	5	53
		**Percentage**	**95% CI**	**Percentage**	**95% CI**	**Percentage**	**95% CI**
	**Sensitivity**	72.73	49.78 to 89.20	100.00	89.90 to 100.00	96.75	92.58 to 98.93
	**Specificity**	100.00	98.01 to 100.00	98.84	95.85 to 99.83	100.00	93.21 to 100.00
	**PPV**	100.00	79.24 to 100.00	94.59	81.77 to 99.18	100.00	97.53 to 100.00
	**NPV**	96.86	93.28 to 98.83	100.00	97.83 to 100.00	91.38	81.01 to 97.11
	**DP**	10.63	6.78 to 15.65	16.91	12.07 to 22.72	74.40	67.88 to 80.19

*Included two triple-species mixed infections and five none speciation samples.

## Discussion

PlasmoNex™ is a conventional multiplex PCR system developed for the simultaneous identification and differentiation of all five human malaria parasites in a single tube reaction together with an internal control. The system showed to be of high accuracy (sensitivity and specificity) in identification and differentiation of all five human *Plasmodium* species in both single- and mixed-species infections [9] and is applicable for usage in epidemiological study [17]. The real-time PCR applied in the present study was adapted from three published studies [10,12,14]. The genus-conserved primers, i.e., Plasmo1 and Plasmo2 and Plasprobe used to detect the presence of *Plasmodium* species originated from Rougemont *et al.* [10]. In their study, four major human *Plasmodium* species-specific probes, i.e., Falcprobe, Vivprobe, Ovaprobe, and Malaprobe were developed in order to further discriminate malaria parasites up to species level [10]. Basically, species-specific real-time PCR described by Rougemont *et al.* was designed to simultaneously identify all four species in two separate multiplex PCR mixtures, i.e., Falcprobe multiplexed with Vivprobe and Malaprobe multiplexed with Ovaprobe. The Pk probe specific for *P. knowlesi* detection was then developed in complementary to this *Plasmodium* screening assay [14]. One of the limitation of Rougemont *et al.* method is the inability of the assay to detect mixed infections, which is likely due to competition of the conserved primers (Plasmo1 and Plasmo2) for the different templates and biasness in amplification of species with higher level of infection [12,18]. Several years later, Shokoples *et al.* improved this method by using a set of specific-specific forward primers targeting four major *Plasmodium* species (excluding *P. knowlesi*) in replacement of genus-conserved forward primer (Plasmo1). In combination with a conserved reverse primer (Plasmo2) and species-specific probes, this real-time PCR assay was optimized for the multiplex assay in a single tube reaction, which also included the careful validation of single- and mixed-species infections [12]. Generally, there are several advantages of real-time PCR over conventional PCR. The real-time PCR is considered a rapid assay and the result is obtained in a straightforward manner based on completion of amplification without any post-PCR downstream analysis such as gel electrophoresis for result interpretation. The ability in quantification of DNA copy number as correlated with parasites density by microscopic examination which cannot be achieved by conventional PCR approaches is the major strength of the real-time PCR assays; however, the cost of reagents and equipment are much higher than that of any conventional PCR assays. Quantitative analysis of parasitaemia by real-time PCR does correspond with the clinical presentation of the disease and is useful in post-treatment detection of *Plasmodium* DNA to monitor response to therapy and/or to predict treatment failure possibly due to parasite resistance [19].

In the present study, no diagnostic divergence was assumed in the experiment design as the DNA samples were from the same source and both PCR assays described here were targeting *Plasmodium* 18S ssu rRNA gene. All DNA samples used here were successful extracted as indicated by the presence of internal positive control band (i.e., human β-haemoglobin in PlasmoNex™ assay) and fluorescence signal (i.e., human β2-macroglobulin in real-time PCR).

The PlasmoNex™ PCR assay is the only multiplex system that allows simultaneous identification and differentiation of all five human *Plasmodium* parasites in a single tube reaction. The accuracy of the assay was also being observed in the present study, in which two triple-species mixed infections were successful diagnosed and further confirmed by sequencing data. Of the 207 infected samples, nine had discrepant species identification based on two PCR assays. Two samples with *P. falciparum* positive as determined by real-time PCR were actually *P. knowlesi* single-species infection determined by PlasmoNex™ and confirmed by DNA sequencing. Five *Plasmodium* positive (Plasprobe positive) samples, which failed to be determined up to species level (species-specific probes negative) by real-time PCR were actually single infection of *P. vivax* (n = 4) and *P. knowlesi* (n = 1) based on PlasmoNex™ assay and sequencing results. The major finding in the present study was that the species-specific real-time PCR did not seem to be as specific as PlasmoNex™ assay especially in the detection of mixed infections. In two samples with triple-species infections by *P. vivax*, *P. falciparum*, and *P. knowlesi*, multiplex real-time PCR (for *P. vivax*, *P. falciparum*, and *P. malariae*) and monoplex real-time PCR (for *P. knowlesi*) only successful picked up the *P. falciparum* infection. Failure of the multiplex real-time PCR indicated that there were possibly some internal diagnosis constraints, maybe due to competition for genus-conserved reverse primer or PCR reagents. Inter-laboratory variation such as difference in PCR reagent used, source of hydrolysis probes, type of thermocycler used, etc. might also be the contributing factors (not tested here). The failure of monoplex real-time PCR in the determination of *P. knowlesi* in cases with mixed infections can be explained by the possibility of diagnostic constraint present in the real-time PCR as commented on the real-time PCR developed by Rougemont *et al.* [12,18]. The Plasmo1 and Plasmo2 adopted in monoplex real-time PCR for *P. knowlesi* detection are genus-conserved primers for all five human *Plasmodium* species, therefore in the cases of mixed infections, *P. falciparum* and *P. vivax* fragments may also be co-amplified with the *P. knowlesi* fragment and this certainly lowered the concentration of *P. knowlesi* amplicon, possibly to the level below the threshold of Pk probe. Furthermore, in the mixed infections, parasite densities are varied substantially and there is a possibility of biasness in the amplification of the species with high loads. In contrast, this diagnostic constraint (primer competition) was not observed in PlasmoNex™ assay, because the sensitivity and specificity of the hexaplex PCR assay were tested empirically to all five human *Plasmodium* species. From the results, the sensitivity and specificity of multiplex real-time PCR utilized in the present study seem to be limited especially in the cases of mixed infections. Furthermore, this real-time PCR assay was optimized and tested on four human *Plasmodium* species excluding *P. knowlesi*. Further validation on the sensitivity and specificity of the assay are needed prior to recruiting this assay as a routine malaria diagnostic tool.

In Malaysia, *P. knowlesi* is recognized as a common cause of severe and potentially fatal human malaria. To date, 19 knowlesi malaria deaths have been reported in Malaysia Borneo, 12 cases in Sabah state [20,21] and seven cases in Sarawak [22-24], confirmed by PCR. Again, 72% of the malaria cases caused by *P. knowlesi* were confirmed using both molecular approaches in this study (Table 2). This further emphasizes the necessity to include molecular specific assay for *P. knowlesi* diagnosis as well as in surveillance and epidemiological studies.

## Conclusions

Malaria is predominantly widespread in the tropical and subtropical regions and exerts immense health and economic burdens in many economic disadvantaged countries. Microscopic examination is the global accepted gold standard for routine laboratory diagnostic method for malaria. The invention of molecular PCR diagnostic tools can be useful for prospective and retrospective analysis of samples for surveillance and epidemiological studies. Of the currently available PCR assays, a straightforward single step multiplex PCR speed up the time for results compared with conventional molecular gold standard nested PCR assay. The PlasmoNex™ PCR assay seems to be more accurate than species-specific real-time PCR in the identification and differentiation of all five human malaria parasites up to species level in single- as well as mixed-species infections. This assay has successfully detected two triple-species mixed infections, which were misdiagnosed as *P. falciparum* single-species infection by real-time PCR. This suggests that PlasmoNex™ PCR assay may serve as an ideal adjunct method for accurate and effective diagnosis of patients presenting with malaria symptoms. The present study again provide evidences that *P. knowlesi* infections appear to be on the increasing trend, with the species now accounting for the majority of malaria cases in Sabah state after the state successful controlled malaria caused by *P. falciparum* and *P. vivax*. The increasing number of *P. knowlesi* infection that can be potentially lethal is now not only widespread in Malaysia but there is also a trend of emergence in many other countries of Southeast Asia. The growing impact of ecotourism and economic development in Malaysia are expected to subsequently lead to further increase in cases among locals and among travellers. Clinicians and laboratory personnel should be alert of this emerging species because it can be confused with benign *P. malariae* when diagnosed solely by microscopy.

## References

[1] World Health Organization (2013). World malaria report 2013.

[2] Rundi C. Malaria elimination in Malaysia [http://apmen.org/storage/apmen-iii/Dr%20Christina%20Rundi.pdf]

[3] Moody A (2002). Rapid diagnostic tests for malaria parasites. Clin Microbiol Rev.

[4] Singh B, Bobogare A, Cox-Singh J, Snounou G, Abdullah MS, Rahman HA (1999). A genus- and species-specific nested polymerase chain reaction malaria detection assay for epidemiologic studies. Am J Trop Med Hyg.

[5] Singh B, Kim Sung L, Matusop A, Radhakrishnan A, Shamsul SS, Cox-Singh J (2004). A large focus of naturally acquired *Plasmodium knowlesi* infections in human beings. Lancet.

[6] Snounou G, Viriyakosol S, Zhu XP, Jarra W, Pinheiro L, Do Rosario VE (1993). High sensitivity of detection of human malaria parasites by the use of nested polymerase chain reaction. Mol Biochem Parasitol.

[7] Rubio JM, Benito A, Roche J, Berzosa PJ, Garcia ML, Mico M (1999). Semi-nested, multiplex polymerase chain reaction for detection of human malaria parasites and evidence of *Plasmodium vivax* infection in Equatorial Guinea. Am J Trop Med Hyg.

[8] Padley D, Moody AH, Chiodini PL, Saldanha J (2003). Use of a rapid, single-round, multiplex PCR to detect malarial parasites and identify the species present. Ann Trop Med Parasitol.

[9] Chew CH, Lim YAL, Lee PC, Mahmud R, Chua KH (2012). Hexaplex PCR detection system for identification of five human *Plasmodium* species with an internal control. J Clin Microbiol.

[10] Rougemont M, Van Saanen M, Sahli R, Hinrikson HP, Bille J, Jaton K (2004). Detection of four *Plasmodium* species in blood from humans by 18S rRNA gene subunit-based and species-specific real-time PCR assays. J Clin Microbiol.

[11] Mangold KA, Manson RU, Koay ES, Stephens L, Regner M, Thomson RB (2005). Real-time PCR for detection and identification of *Plasmodium* spp. J Clin Microbiol.

[12] Shokoples SE, Ndao M, Kowalewska-Grochowska K, Yanow SK (2009). Multiplexed real-time PCR assay for discrimination of *Plasmodium* species with improved sensitivity for mixed infections. J Clin Microbiol.

[13] Han ET, Watanabe R, Sattabongkot J, Khuntirat B, Sirichaisinthop J, Iriko H (2007). Detection of four *Plasmodium* species by genus- and species-specific loop-mediated isothermal amplification for clinical diagnosis. J Clin Microbiol.

[14] Divis PC, Shokoples SE, Singh B, Yanow SK (2010). A TaqMan real-time PCR assay for the detection and quantitation of *Plasmodium knowlesi*. Malar J.

[15] Watzinger F, Suda M, Preuner S, Baumgartinger R, Ebner K, Baskova L (2004). Real-time quantitative PCR assays for detection and monitoring of pathogenic human viruses in immunosuppressed pediatric patients. J Clin Microbiol..

[16] Zweig MH, Campbell G (1993). Receiver-operating characteristic (ROC) plots: a fundamental evaluation tool in clinical medicine. Clin Chem.

[17] Goh XT, Lim YA, Vythilingam I, Chew CH, Lee PC, Ngui R (2013). Increased detection of *Plasmodium knowlesi* in Sandakan division, Sabah as revealed by PlasmoNex. Malar J.

[18] Bialasiewicz S, Whiley DM, Nissen MD, Sloots TP (2007). Impact of competitive inhibition and sequence variation upon the sensitivity of malaria PCR. J Clin Microbiol.

[19] Kain KC, Kyle DE, Wongsrichanalai C, Brown AE, Webster HK, Vanijanonta S (1994). Qualitative and semiquantitative polymerase chain reaction to predict *Plasmodium falciparum* treatment failure. J Infect Dis.

[20] William T, Menon J, Rajahram G, Chan L, Ma G, Donaldson S (2011). Severe *Plasmodium knowlesi* malaria in a tertiary care hospital, Sabah, Malaysia. Emerg Infect Dis.

[21] Rajahram GS, Barber BE, William T, Menon J, Anstey NM, Yeo TW (2012). Deaths due to *Plasmodium knowlesi* malaria in Sabah, Malaysia: association with reporting as Plasmodium malariae and delayed parenteral artesunate. Malar J.

[22] Cox-Singh J, Davis TM, Lee KS, Shamsul SS, Matusop A, Ratnam S (2008). *Plasmodium knowlesi* malaria in humans is widely distributed and potentially life threatening. Clin Infect Dis.

[23] Daneshvar C, Davis TM, Cox-Singh J, Rafa'ee MZ, Zakaria SK, Divis PC (2009). Clinical and laboratory features of human *Plasmodium knowlesi* infection. Clin Infect Dis.

[24] Cox-Singh J, Hiu J, Lucas SB, Divis PC, Zulkarnaen M, Chandran P (2010). Severe malaria - a case of fatal *Plasmodium knowlesi* infection with post-mortem findings: a case report. Malar J.

